# Understanding Canadians’ knowledge, attitudes and practices related to antimicrobial resistance and antibiotic use: Results from public opinion research

**DOI:** 10.14745/ccdr.v48i1112a08

**Published:** 2022-11-03

**Authors:** Anna-Louise Crago, Stéphanie Alexandre, Kahina Abdesselam, Denise Gravel Tropper, Michael Hartmann, Glenys Smith, Tanya Lary

**Affiliations:** 1 Antimicrobial Resistance Task Force of the Public Health Agency of Canada

**Keywords:** antimicrobial resistance, antibiotic resistance, antibiotic use, public opinion, survey, Canada

## Abstract

**Background:**

Antimicrobial resistance is a current and pressing issue in Canada. Population-level antibiotic consumption is a key driver. The Public Health Agency of Canada undertook a comprehensive assessment of the Canadian public’s knowledge, attitudes and practices in relation to antimicrobial resistance and antibiotic use, to help inform the implementation of public awareness and knowledge mobilization.

**Methods:**

Data were collected in three phases: 1) six in-person focus groups (53 participants) to help frame the survey; 2) nationwide survey administration to 1,515 Canadians 18 years and older via cell phone and landline; and 3) 12 online focus groups to analyze survey responses. Survey data is descriptive.

**Results:**

A third (33.9%) of survey respondents reported using antibiotics at least once in the previous 12 months, 15.8% more than twice and 4.6% more than five times. Antibiotic use was reported more among 1) those with a household income below $60,000, 2) those with a medical condition, 3) those without a university education and 4) among the youngest adults (18–24 years of age) and (25–34 years of age). Misinformation about antibiotics was common: 32.5% said antibiotics “can kill viruses”; 27.9% said they are “effective against colds and flu”; and 45.8% said they are “effective in treating fungal infections”. Inaccurate information was reported more often by those 1) aged 18–24 years, 2) with a high school degree or less and 3) with a household income below $60,000. In focus groups, the time/money trade-offs involved in accessing medical care were reported to contribute to pushing for a prescription or using unprescribed antibiotics, particularly in more remote contexts, while the cost of a prescription contributed to sharing and using old antibiotics. A large majority, across all demographic groups, followed the advice of medical professionals in making health decisions.

**Conclusion:**

High trust in medical professionals presents an important opportunity for knowledge mobilization. Delayed prescriptions may alleviate concerns about the time/money constraints of accessing future care. Consideration should be given to prioritizing access to appropriate diagnostic and other technology for northern and/or remote communities and/or medical settings serving many young children to alleviate concerns of needing a prescription or of needing to return later.

## Introduction

Antimicrobial resistance (AMR) is a current and pressing issue in Canada, though information on more benign infections is limited, some calculations estimate that as many as 26% of infections may be resistant to first line antimicrobials ((1)). In Canada, AMR is estimated to cause 15 deaths a day and cost $1.4 billion dollars a year ((1)). Population-level antibiotic consumption is a key driver of AMR ((2)). Assessing the Canadian public’s knowledge, attitudes and practices (KAP) related to antibiotics can help identify barriers to curbing antibiotic use, offer insight into consumption practises and provide a baseline for assessing different interventions.

In 2008, the Public Health Agency of Canada collected a small amount of data on KAP relating to antibiotics as part of a larger public opinion survey on pathogens and infection control ((3)). This was followed, in 2018, by a rapid response module from Statistics Canada’s 2018 community health survey that gathered data specifically on oral antibiotic use ((4)). To have both a current and more comprehensive assessment of the Canadian public’s KAP as they relate to AMR and antibiotics, the Public Health Agency of Canada undertook public opinion research between 2019 and 2022. The data from this research will be used to inform the *Pan-Canadian Action Plan on Antimicrobial Resistance* and to target stewardship and awareness activities.

## Methods

Researchers from The Strategic Counsel collected data in three phases. In-person focus groups were held in July 16–18, 2019 to gather preliminary insights into KAP related to antibiotics and AMR, to frame the survey questionnaire. Participants were divided into six focus groups representing different gender and age categories; each group had a cross-section of different employment statuses, household incomes and ethnicities. This phase was followed by the development of a 19-minute-long telephone survey on AMR and antibiotic KAP adhering to the *Standards for the Conduct of Government of Canada Public Opinion Research—Telephone surveys* ((5)). The survey was pre-tested in both official languages (English and French) among 20 respondents on December 7, 2021, and the overwhelming majority of respondents (95%) reported the questionnaire was easily understood. The survey was administered nationwide to 1,515 Canadians 18 years of age and older, via cell phone and landline (60/40 split) between December 10, 2021, and January 7, 2022. Participants were informed that the survey data was for the Public Health Agency of Canada and that their participation was voluntary and confidential.

The survey broadly covered three areas: knowledge and perception of antibiotics; antibiotic use and health practises; and knowledge, awareness and perception of AMR. It included standard public opinion research questions on antibiotic use and familiarity with terms. It also included questions on health decision-making strategies more broadly to identify the most impactful circumstances for education on antibiotics and AMR.

A stratified sample design was utilized to ensure sufficient data from Saskatchewan, Manitoba and the Atlantic provinces for the possibility of regional comparisons for future analyses. Nationally, the results have an associated margin of error of (+/-) 2.5%, at a 95% confidence level. Results for population subgroups have a higher associated margin of error. All percentages reported are based on the weighted sample. Descriptive analyses of the survey data were done using SAS software 9.4 (SAS Institute; Cary, United States).

The telephone survey took place while the Omicron wave of coronavirus disease 2019 (COVID-19) was rampant in most parts of the country. Questions referring to the prior 12 months refer to a period when COVID-19 was prevalent and there were associated public health measures in many areas. The anomalous circumstances of this period appear to have impacted at least some facets of antibiotic use. Data on subscription of systemic antibiotics shows a decline in community antibiotic use in 2020 and 2021 beginning at the onset of COVID-19 ((6)). We do not have data specifically on unprescribed, non-systemic or over the counter use during this period.

The third phase consisted of 12 online focus groups (held between February 23 and March 1, 2022), whose participants were recruited from both urban centres and more rural and northern communities to probe more deeply into attitudes and behaviours linked to antibiotics and AMR. Focus groups used a moderated round-table discussion format following a set moderator guide and touched on three subject areas: knowledge and awareness of antibiotics; antibiotic use; and knowledge and awareness of antimicrobial resistance. A qualitative approach allowed for a more in-depth exploration of mindset, motivations, barriers, and personal or social considerations as they related to these issues. Participants were again divided into groups representing different gender and age categories, each with a cross-section of different employment statuses, household incomes and ethnicities. Additionally, some groups were restricted to parents of young children, Indigenous or Asian-Canadian participants to ensure representation of their views. A preliminary analysis of themes reported in the focus groups was performed by The Strategic Counsel and these were subsequently analyzed for cross-cutting themes related to antibiotic use.

## Results

### Participants, response rate and sample of telephone survey

There were 53 participants in the in-person focus groups (phase 1) and 101 participants in the on-line focus groups (phase 3). In total, 1,515 respondents completed the telephone survey, with a completion rate of 99.62%. The overall response rate was 2.77% calculated using the empirical method formula of R/(U + IS + R). There were 1,583 responding (R) participants (completed, disqualified and over-quota respondents), 44,436 unresolved numbers (U) and 11,283 in scope non-responding participants (IS).

The demographics of all respondents (both weighted and unweighted) are summarized in **Table 1**.

**Table 1:** Demographics of respondents

Respondent demographics	Respondents, N=3,015		
	Weighted, n=1,500		Unweighted, n=1,515
	n	%	
Gender			
Male	723	48.2	697
Female	764	50.9	808
Other	13	0.9	10
Age group			
18–24 years	163	10.9	95
25–34 years	244	16.2	209
35–44 years	242	16.2	234
45–54 years	266	17.8	241
55–64 years	260	17.3	285
65 years and older	314	21.0	440
Prefer not to answer	11	0.7	11
Education			
High school or less	375	25.0	393
College/trades	389	25.9	398
University	720	48.0	708
Prefer not to answer	16	1.1	16
Income			
Less than $60,000	477	31.8	498
$60,000 to less than $100,000	364	24.3	361
$100,000 or more	446	29.8	432
Prefer not to answer	213	14.2	224
Language			
English	1,027	68.5	1,047
French	312	20.8	321
Other	155	10.3	141
Prefer not to answer	5	0.4	6
Medical condition			
Yes	383	25.6	362
No	1,109	74.0	1,145
Prefer not to answer	7	0.5	8

### Knowledge of antibiotics

**Figure 1:**
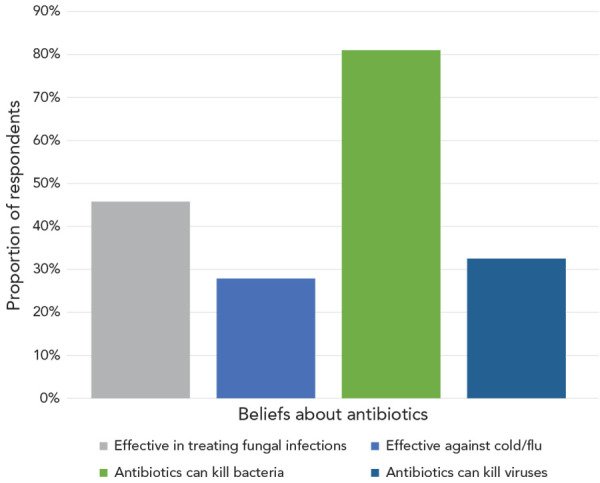
Knowledge of antibiotics among respondents

More than three quarters (81.0%) of survey respondents correctly identified that antibiotics “can kill bacteria”; however, many respondents were misinformed about many other elements of antibiotic use and misuse. Nearly a third (32.5%) said that antibiotics “can kill viruses” or that they are “effective against colds and flu” (27.9%). Almost half (45.8%) said they “are effective in treating fungal infections” (**Figure 1**).

Inaccurate information on antibiotics’ effectiveness against viruses, colds and flu and fungal infections was consistently reported more often by those aged 18–24 years (41.9%, 54.7%, 58.0%, respectively), those with a high school degree or less (45.0%, 41.4%, 54.2%, respectively) and those with a household income below $60,000 (41.3%, 36.7%, 51.9%, respectively). Those who spoke French at home were more likely to report effectiveness against viruses (42.9%) and fungal infections (53.7%), while those who spoke neither English or French at home were more likely to report that they were effective against colds and flu (41.2%).

### Antibiotic use

Slightly more than a third (33.9%) of survey respondents reported using antibiotics at least once in the past 12 months: 15.8% had used antibiotics more than twice in the past 12 months; and 4.6% had used antibiotics more than five times in the past 12 months. The questions in this survey cover all antibiotic use regardless of format (e.g. pill, injection, topical), mechanism of action (e.g. systemic or local) and means of access (prescribed, unprescribed over the counter).

**Figure 2:**
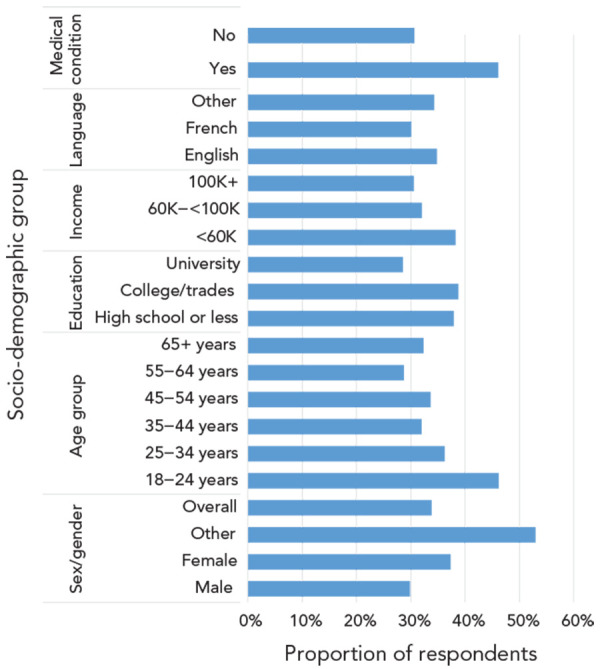
Reported antibiotic use in past 12 months by socio-demographic variable

Antibiotic use was reported more among those with a medical condition (46.1%), young adults (18–24 years of age, 46.2%; 25–34 years of age, 36.3%), those with a household income below $60,000 (38.2%) and those without a university education (38.8% for those with college or trades and 37.9% for those with high school). Slightly more women (37.3%) than men (29.7%) reported using antibiotics (**Figure 2**). Similarly, frequent use (more than twice in the past 12 months) was reported more by those with a medical condition (26.9%), by the youngest adults (18–24 years of age, 25.2%), by those with a household income below $60,000 (21.7%) and by those with high school or less (21%) or college/trade diplomas (20.6%) (**Table 2**).

**Table 2:** Reported antibiotic use by socio-demographic variables and by frequency in the previous 12 months

Socio-demographic variables	Once		2–5 times		5 or more times		Never/none		Don’t know/refused to answer	
	n	%	n	%	n	%	n	%	n	%
Medical condition										
Yes	61	19.2	50	15.9	35	10.9	165	52.3	5	1.6
No	209	17.8	116	9.9	35	3.0	803	68.5	10	0.8
Language										
English	193	18.8	122	11.9	42	4.1	656	63.8	14	1.4
French	47	15.0	26	8.4	21	6.7	218	69.9	N/A	N/A
Other	32	20.4	17	11.3	4	2.6	101	65.2	1	0.5
Income										
Less than $60,000	79	16.5	73	15.3	31	6.5	290	60.8	5	0.9
$60,000 to less than $100,000	66	18.2	32	8.8	19	5.1	242	66.5	5	1.4
$100,000 or more	88	19.8	39	8.8	9	1.9	309	69.3	1	0.1
Education										
High school or less	64	17.0	58	15.4	21	5.6	227	60.5	6	1.6
College/trades	71	18.3	51	13.2	28	7.2	234	60.2	4	1
University	134	18.6	55	7.6	17	2.4	509	70.8	5	0.7
Age group										
18–24 years	34	21.0	31	19.2	10	6.0	88	53.8	0	0.0
25–34 years	41	16.8	31	12.7	17	6.8	152	62.4	3	1.4
35–44 years	54	22.4	16	6.8	7	2.8	164	67.8	1	0.2
45–54 years	51	19.3	26	9.8	12	4.6	177	66.4	0	0.0
55–64 years	46	17.9	22	8.5	6	2.3	181	69.8	4	1.5
65 years and older	44	14.1	40	12.6	18	5.6	206	65.7	6	2.0
Gender										
Male	109	15.1	80	11.0	26	3.6	497	68.8	10	1.4
Female	158	20.7	86	11.2	41	5.4	475	62.1	4	0.5
Total	271	18.1	168	11.2	69	4.6	977	65.1	15	1.0

### Strategies for health decision-making

**Figure 3:**
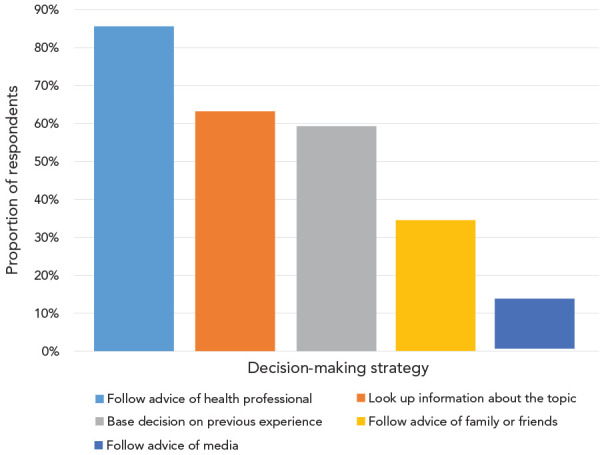
Decision-making - strategies reported among different groups of respondents

Respondents reported three main strategies for making health decisions in general (multiple answers were permitted). A large majority (85.6%) indicated that they follow the advice of a health professional, almost two thirds report searching for relevant information themselves (63.3%) or relying on their previous experience (59.3%) (**Figure 3**).

**Figure 4:**
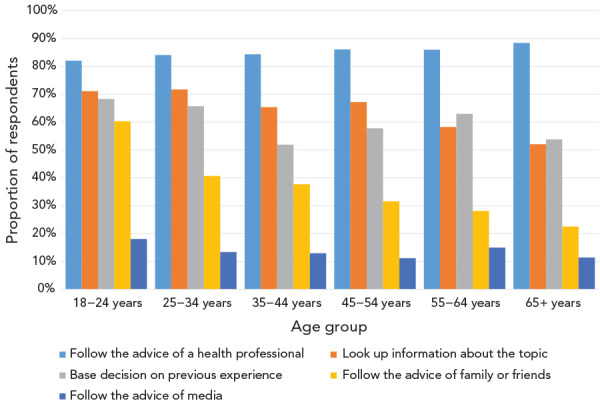
Decision-making strategies by age group

Women were more likely than men to report following the advice of a health professional (89.3% vs 81.9%). There were very high reported levels of following the advice of a health professional, irrespective of household income, education level, age, or language spoken. Younger respondents were more likely to report looking up health information themselves, to base their decision on their previous experience and/or to follow the advice of family or friends compared with older respondents (**Figure 4**).

### Factors shaping antibiotic use: cross-cutting themes from focus groups

Two cross-cutting themes emerged out of the focus groups related to factors shaping antibiotic practices. The first was the role of difficulties accessing primary care and the time/money trade-offs involved in going to the doctor. Many respondents disclosed that they shared antibiotics or wanted to get an antibiotic prescription when they saw a health professional because of the difficulties of accessing care or of being able to return to get a prescription later if eventually needed. Women in a focus group with high Indigenous representation noted it was common practice in their communities to keep some antibiotic from a prescription in case those were needed in the future, due to the lack of access to a doctor.

Another cross-cutting theme was the cost of prescriptions and resultant financial pressures on families. This was cited as a reason for sharing prescriptions or keeping old pills. It was also the main reason cited by a small number of respondents for purchasing large quantities of antibiotics abroad, where they were available over the counter, for their children’s eventual use in Canada.

### Knowledge and attitudes related to antibiotic/antimicrobial resistance

**Figure 5:**
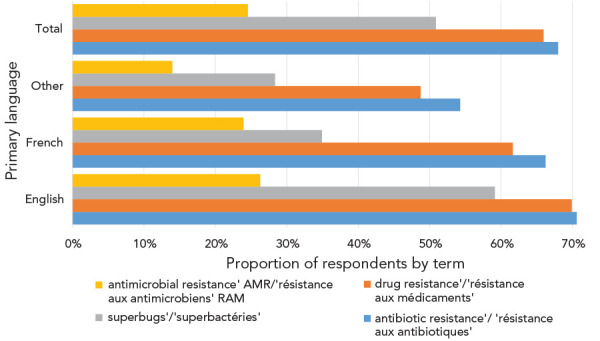
Knowledge of terms by primary language spoken at home Abbreviations: AMR, antimicrobial resistance; RAM, résistance aux antimicrobiens

Approximately, a quarter of Canadians polled (24.6%) reported knowing the term “antimicrobial resistance” / “résistance aux antimicrobiens”, 68.0% knew “antibiotic resistance” / “résistance aux antibiotiques” and 66.0% knew “drug resistance” / “résistance aux médicaments”. Half (50.9%) of respondents were familiar with “superbugs” / ”superbactéries”—these terms were only known to a majority of people who spoke English at home (**Figure 5**).

Nearly a quarter (22.0%) reported that they or someone they knew had experienced antibiotic resistance, while 8.4% reported that they or someone they knew had experienced antimicrobial resistance. This discrepancy is most likely due to lower familiarity with the term “antimicrobial” as compared with “antibiotic”. In focus groups, a theme that emerged was that many people did not feel AMR was an issue that affected them or their families directly.

Once provided with an explanation of AMR, a majority (57.5%) expressed concern: 41.5% were “somewhat worried” and 16.0% were “very worried”. In focus groups, AMR was not necessarily seen as a “top 10” global public health threat nor viewed as a particularly urgent issue. Concern about AMR was slightly higher among those with a university education (62.2%), those who spoke French at home (62.4%) and those aged 55–64 years (62.1%).

## Discussion

The results reported here are quite similar to those reported in 2008, which were based on a nationwide sample of 1,500 participants, a representative sample of the Canadian population at the time ((3)). The proportion of Canadians reporting antibiotic use in the prior 12 months has declined slightly, from 38% to 34%, in the past 14 years. A slightly higher proportion of respondents now incorrectly reports that antibiotics are effective against “colds and flu” (28%) than those that reported they were effective against “colds” in 2008 (24%). Concern about resistance to antibiotics has declined slightly since 2008—from 59% to 57% ((3)). These differences may fall within the combined margins of error for both surveys (2.4% in 2008 and 2.5% in 2022). A slightly lower proportion of Canadians now incorrectly reports that antibiotics kill viruses (39% in 2008 vs. 33% vs in 2022) ((3)).

Regarding knowledge of antibiotics and antimicrobial resistance, further research might help clarify whether misinformation is rooted in a conflation of antiviral or antifungal medication with antibiotics, a misunderstanding of the different kinds of pathogens that can cause infection or a lack of clarity on antibiotics’ scope of action. A more refined understanding of the sources of misinformation could assist in targeting education efforts. The large gap between the proportion of respondents reporting familiarity with the terms “drug resistance” (66.0%) and “antibiotic resistance” (68.0%) versus “antimicrobial resistance” (24.6%) is important to keep in mind for public education efforts as public education efforts increasingly move towards the latter language. When the concept is explained, Canadians report much lower concern about antimicrobial resistance (57%) when compared to other high-income countries such as the United States (81%) ((7)) and the United Kingdom (88%) ((8)). Canadians report a similar level of incorrect information on antibiotics killing viruses as people in the United Kingdom (33% and 28%, respectively) and a similar level of antibiotic use (34% and 33%, respectively) ((8)).

In this study, people with lower income levels had much higher frequent use of antibiotics than their peers. Multiple factors may contribute to this observation. This may be driven by a high burden of medical conditions in lower-income communities in Canada ((9)), including infections ((10)). Antibiotic use may be linked to lower vaccination rates with various vaccines in low-income communities ((11,12)). Those with household incomes below $60,000 also had lower levels of knowledge about antibiotic use; however, individuals with low incomes and low education levels both expressed high trust in doctors as a source of health information and a large majority followed medical professionals’ advice in making health decisions, presenting an important opportunity for stewardship interventions.

Young adults (18–34 years of age) reported use, and in particular frequent use, of antibiotics—far more than other age groups. Our findings likely underestimate use among the elderly due to the use of a broad older age category (65 years of age and older) and under-sampling of the very elderly who may be more dependent on caregivers or living in hospitals or long-term care. It is possible that higher levels of misinformation on antibiotics among young adults (18–24 years of age) led to overreporting of antibiotic use in the youngest age group, though depending on the mistaken underlying belief, it could also be consistent with high use. As well, due to higher margins of error among subgroups, these differences may not be significant or may fall within the margin of error. High levels of reported use among young adults are nonetheless consistent with findings from the 2018 Canadian Community Health Survey (CCHS) and those from public opinion research in Québec. Indeed, CCHS (25,787 participants over 18 years of age from all provinces, weighted to be representative) found a high frequency of specifically oral antibiotic use reported in this age group ((4)) while public opinion research in Québec (a representative sample of 7,254 participants) found that 25–34 year-olds had the highest reported levels of antibiotic use ((13)). Young adults were also more likely to have recent prescriptions in the 2008 nationwide survey ((3)). In contrast, national surveillance data on antibiotic dispensation according to tonnage (defined daily doses) and according to the overall number of prescriptions per 1,000 inhabitants show levels rising with age ((14)). This discrepancy may be due to the latter data excluding non-systemic antibiotics (such as creams, gels, vaginal tablets, eye drops and other formats), which can be used to treat some infections that are found disproportionately in young adults, to different metrics that are difficult to compare directly or to the inability of surveillance data to capture unprescribed use, which may be higher in young adults.

Young adults are also frequently the parents of young children and are an important group to consider for health promotion; however, initiatives need to be tailored to respond to specific use patterns and challenges. The youngest adults (18–24 years of age) report more incorrect information on appropriate antibiotic use than older age groups. Adults younger than 35 years of age were more likely to make health decisions based on their previous experience, by following the advice of family or friends or by looking up health information themselves in comparison with older age groups. They are also more vulnerable to health misinformation ((15)). Finally, young adults, and in particular young men, are among the groups with the highest vaccine hesitancy or opposition in Canada ((12)), with the lowest rates of vaccination for the flu and for three or more doses of the COVID-19 vaccine ((11,16)). This is a concern given the effectiveness of vaccination as a strategy for reducing antibiotic use ((17)).

The focus group's findings echo previous research that identified the challenges of accessing care and the time/money trade-offs involved in doing so as factors in understanding antibiotic use, particularly in relation to gendered care burdens ((18)). Concerns about time/money trade-offs are also specifically associated with unprescribed use in other studies ((19)). This report illustrates that this issue may particularly affect remote and/or Indigenous communities. These findings provide insight into the 2008 public opinions research results that almost twice as many northern residents reported that their most recent antibiotic was from an old prescription as compared with other Canadians (14% vs 8%, respectively) ((3)). High rates of use in some Northern Indigenous communities are attributed to the high burden of infections, lack of access to physician care and lack of diagnostic capabilities ((20)).

## Limitations and strengths

There are several limitations to this study. Data are self-reported and subject to recall bias and response bias. Respondents may not have understood certain terms in the questions. Any survey may contain potential errors such as coverage and measurement errors. The response rate was consistent with very low response rates for telephone surveys in recent years, following a two decade declining trend ((21,22)). In 2018, the Pew Center found the average response rate for telephone surveys was 6% ((21)). Low response rates can introduce greater nonresponse bias; however, a number of studies have found that response rates are not strongly associated with accuracy ((21-23)).

Telephone surveys exclude vulnerable populations, such as institutionalized and homeless populations, as well as populations that may not have a phone due to low incomes or precarity. Telephone surveys may also exclude people who are not well enough to respond or who are dependent on a caregiver for phone access; this may disproportionately exclude the elderly and/or disabled.

An important limitation is that this data set can only be used for descriptive purposes. Additionally, results are not disaggregated by racialized group, ethnic group and/or Indigenous status, and the sex/gender category of “other” has too few respondents to be able to meaningfully interpret results. Lastly, this survey did not collect disaggregated data specifically on prescribed, unprescribed, or over-the-counter use.

A strength of this research is the breadth of antibiotic use that it captures. It is one of the only current data streams in Canada to include unprescribed use and non-systemic use. This allows important insight into how common antibiotic use is, which is an important consideration for any awareness or education effort.

## Conclusion

This public opinion research offers insight into the general population’s knowledge, attitudes and practices with regards to antibiotics and AMR, helping to shape and inform efforts to address AMR reduction initiatives for the general population. Gaps remain in knowledge on how to support health promotion and stewardship in high-risk environments for AMR in the community, such as long-term care facilities and prisons, and with key populations at higher risk or with a higher burden of community-acquired resistant pathogens. Further studies using electronic medical records and studies on unprescribed use and over-the-counter use can shed light on some of the discrepancies between public opinion research findings and antibiotic dispensing data and help us better understand patterns of use in different demographics.

High trust in medical professionals and reported adherence to medical advice presents an important opportunity for reaching populations reporting high levels of antibiotic use and holding incorrect information frequently, such as young adults and those in low-income households. Findings from research on vaccine hesitancy have similarly identified medical providers as playing a key role as trusted and persuasive sources of medical advice ((24-31)) and, of relevance to medical provider interventions regarding antibiotic use and AMR. These studies have found that the most effective interventions include clear information on both individual and community risks and benefits ((25)) and direct medical recommendations ((24-31)).

As well, delayed prescriptions—prescriptions made available at a later date if symptoms persist in a way consistent with bacterial infection—may reduce unnecessary use while alleviating concerns about the time/money constraints of accessing future care. Access to appropriate diagnostic and other technology could be prioritized for Northern, Indigenous and/or remote communities and/or healthcare settings serving many young children to alleviate concerns of needing a prescription or of needing to return later for a prescription.

## References

[1] Council of Canadian Academies. When Antibiotics Fail. Ottawa: The Expert Panel on the Potential Socio-Economic Impacts of Antimicrobial Resistance in Canada. Ottawa, ON: CAA; 2019. https://cca-reports.ca/reports/the-potential-socio-economic-impacts-of-antimicrobial-resistance-in-canada/

[2] Patrick DM, Hutchinson J. Antibiotic use and population ecology: how you can reduce your “resistance footprint”. CMAJ 2009;180(4):416–21. 10.1503/cmaj.080626 19221355 PMC2638037

[3] Public Health Agency of Canada. Canadians’ Knowledge, Attitudes and Behaviour on Pathogens and Infection Control: Final Report. Ottawa, ON: PHAC; 2008. https://books.google.ca/books/about/Canadians_Knowledge_Attitudes_and_Behavi.html?id=weKczQEACAAJ&redir_esc=y

[4] Statistics Canada. Canadian Community Health Survey (CCHS) – Rapid Response January to June 2018 – Antibiotic Medication Use. Ottawa, ON: StatCan; (modified 2018). https://www23.statcan.gc.ca/imdb/p3Instr.pl?Function=assembleInstr&Item_Id=795197&TET=1

[5] Government of Canada. Standards for Conduct of Government of Canada Public Opinion Research—Telephone surveys. Ottawa, ON: Government of Canada; (modified 2020). https://www.tpsgc-pwgsc.gc.ca/rop-por/telephone-eng.html

[6] Public Health Agency of Canada. Canadian Antimicrobial Resistance Surveillance System Report 2021. Ottawa, ON: PHAC. https://www.canada.ca/en/public-health/services/publications/drugs-health-products/canadian-antimicrobial-resistance-surveillance-system-report-2021.html

[7] Infectious Diseases Society of America and Research!America. AMR Survey. 2018. https://www.researchamerica.org/blog/new-survey-more-80-americans-are-concerned-antibiotic-resistance-health-threat

[8] Hawkins O, Scott AM, Montgomery A, Nicholas B, Mullan J, van Oijen A, Degeling C. Comparing public attitudes, knowledge, beliefs and behaviours towards antibiotics and antimicrobial resistance in Australia, United Kingdom, and Sweden (2010-2021): A systematic review, meta-analysis, and comparative policy analysis. PLoS One 2022;17(1):e0261917. 10.1371/journal.pone.0261917 35030191 PMC8759643

[9] Public Health Agency of Canada. the Pan-Canadian Public Health Network, Statistics Canada and the Canadian Institute for Health Information. Health Inequalities Data Tool. Ottawa, ON: PHAC 2022. https://health-infobase.canada.ca/health-inequalities/Indicat

[10] King T, Schindler R, Chavda S, Conly J. Dimensions of poverty as risk factors for antimicrobial resistant organisms in Canada: a structured narrative review. Antimicrob Resist Infect Control 2022;11(1):18. 10.1186/s13756-022-01059-1 35074013 PMC8785485

[11] Kwong JC, Rosella LC, Johansen H. Trends in influenza vaccination in Canada, 1996/1997 to 2005. Health Rep 2007;18(4):9–19. 18074993

[12] Angus Reid Institute. Dwindling group of unvaccinated cite ‘personal freedom’ and ‘health concerns’ as main reasons for dodging the jab. November 3, 2021. https://angusreid.org/canada-unvaccinated-freedom-reasons/

[13] Institut national de santé publique du Québec (INSPQ). Étude sur les connaissance, attitudes et perceptions de la population québécoise sur l’utilisation des antibiotiques. 2019. https://www.inspq.qc.ca/publications/2690

[14] Public Health Agency of Canada. Canadian Antimicrobial Surveillance Systems Report. 2021. https://www.canada.ca/en/public-health/services/publications/drugs-health-products/canadian-antimicrobial-resistance-surveillance-system-report-2021.html

[15] CanCOVID. Issue Note. Misinformation and disinformation in relation to COVID-19. CanCOVID.ca; April 7, 2021. https://cancovid.ca/wp-content/uploads/2021/06/CanCOVID-Issue-Note-misinformation-EN.pdf

[16] Public Health Agency of Canada. COVID-19 vaccination in Canada. Vaccine Coverage. Ottawa, ON: PHAC; 2022. https://health-infobase.canada.ca/covid-19/vaccination-coverage/#a5

[17] Kwong JC, Maaten S, Upshur RE, Patrick DM, Marra F. The effect of universal influenza immunization on antibiotic prescriptions: an ecological study. Clin Infect Dis 2009;49(5):750–6. 10.1086/605087 19624280

[18] World Health Organization. Tackling antimicrobial resistance together: working paper 5.0: Enhancing the focus on gender and equity. Geneva, CH; WHO; 2018. https://www.who.int/publications/i/item/tackling-antimicrobial-resistance-together-working-paper-5.0-enhancing-the-focus-on-gender-and-equity

[19] Grigoryan L, Germanos G, Zoorob R, Juneja S, Raphael JL, Paasche-Orlow MK, Trautner BW. Use of antibiotics without a prescription in the US population: a scoping review. Ann Intern Med 2019;171(4):257–63. 10.7326/M19-0505 31330541

[20] Williams K, Colquhoun A, Munday R, Goodman KJ; CANHelp Working Group. Antibiotic dispensation rates among participants in community-driven health research projects in Arctic Canada. BMC Public Health 2019;19(1):949. 10.1186/s12889-019-7193-3 31307422 PMC6631451

[21] Public Works and Government Services Canada. Public Opinion Research Directorate. Improving Respondent Cooperation for Telephone Surveys. Ottawa, ON: PWGSC; 2019. https://www.tpsgc-pwgsc.gc.ca/rop-por/rapports-reports/telephone/resume-summary-eng.html

[22] Pew Research Center. Keeter S, Hatley N, Kennedy C, Lau A. What Low Response Rates Mean for Telephone Surveys. Washington (DC): PEW Research; 2017. https://www.pewresearch.org/methods/2017/05/15/what-low-response-rates-mean-for-telephone-surveys/

[23] Groves RM. Nonresponse rates and nonresponse bias in household surveys. Public Opin Q 2006;70(5):646–75. 10.1093/poq/nfl033

[24] Oh NL, Biddell CB, Rhodes BE, Brewer NT. Provider communication and HPV vaccine uptake: A meta-analysis and systematic review. Prev Med 2021;148:106554. 10.1016/j.ypmed.2021.106554 33857561

[25] Fisher KA, Nguyen N, Fouayzi H, Singh S, Crawford S, Mazor KM. Impact of A Physician Recommendation on COVID-19 Vaccination Intent Among Vaccine Hesitant Individuals. Patient Educ Couns 2022;S0738-3991(22):00436-0. https://www.sciencedirect.com/science/article/pii/S0738399122004360?via%3Dihub 10.1016/j.pec.2022.09.013 10.1016/j.pec.2022.09.013 PMC9523946 36244947

[26] Opel DJ, Mangione-Smith R, Robinson JD, Heritage J, DeVere V, Salas HS, Zhou C, Taylor JA. The influence of provider communication behaviors on parental vaccine acceptance and visit experience. Am J Public Health 2015;105(10):1998–2004. 10.2105/AJPH.2014.302425 25790386 PMC4566548

[27] Nguyen KH, Yankey D, Lu PJ, Kriss JL, Brewer NT, Razzaghi H, Meghani M, Manns BJ, Lee JT, Singleton JA. Report of health care provider recommendation for COVID-19 vaccination among adults, by recipient COVID-19 vaccination status and attitudes—united States, April–September 2021. MMWR Morb Mortal Wkly Rep 2021;70(50):1723–30. 10.15585/mmwr.mm7050a1 34914669 PMC8675662

[28] Lu PJ, Srivastav A, Amaya A, Dever JA, Roycroft J, Kurtz MS, O’Halloran A, Williams WW. Association of provider recommendation and offer and influenza vaccination among adults aged ≥18 years - United States. Vaccine 2018;36(6):890–8. 10.1016/j.vaccine.2017.12.016 29329685

[29] Gilkey MB, Calo WA, Moss JL, Shah PD, Marciniak MW, Brewer NT. Provider communication and HPV vaccination: the impact of recommendation quality. Vaccine 2016;34(9):1187–92. 10.1016/j.vaccine.2016.01.023 26812078 PMC4944755

[30] Dempsey AF, Pyrzanowski J, Campagna EJ, Lockhart S, O’Leary ST. Parent report of provider HPV vaccine communication strategies used during a randomized, controlled trial of a provider communication intervention. Vaccine 2019;37(10):1307–12. 10.1016/j.vaccine.2019.01.051 30733088

[31] Kornides ML, McRee AL, Gilkey MB. Parents who decline HPV vaccination: who later accepts and why? Acad Pediatr 2018;18 2S:S37–43. 10.1016/j.acap.2017.06.008 29502636 PMC5859546

